# Antimicrobial activity of some ethnomedicinal plants used by Paliyar tribe from Tamil Nadu, India

**DOI:** 10.1186/1472-6882-6-35

**Published:** 2006-10-17

**Authors:** Veeramuthu Duraipandiyan, Muniappan Ayyanar, Savarimuthu Ignacimuthu

**Affiliations:** 1 Entomology Research Institute, Loyola College, Chennai – 600 034, India

## Abstract

**Background:**

Antimicrobial activity of 18 ethnomedicinal plant extracts were evaluated against nine bacterial strains (*Bacillus subtilis*, *Staphylococcus aureus*, *Staphylococcus epidermidis*, *Enterococcus faecalis*, *Escherichia coli*, *Klebsiella pneumonia*, *Pseudomonas aeruginosa*, *Ervinia sp*, *Proteus vulgaris*) and one fungal strain (*Candida albicans*). The collected ethnomedicinal plants were used in folk medicine in the treatment of skin diseases, venereal diseases, respiratory problems and nervous disorders.

**Methods:**

Plants were collected from Palni hills of Southern Western Ghats and the ethnobotanical data were gathered from traditional healers who inhabit the study area. The hexane and methanol extracts were obtained by cold percolation method and the antimicrobial activity was found using paper disc diffusion method. All microorganisms were obtained from Christian Medical College, Vellore, Tamil Nadu, India.

**Results:**

The results indicated that out of 18 plants, 10 plants exhibited antimicrobial activity against one or more of the tested microorganisms at three different concentrations of 1.25, 2.5 and 5 mg/disc. Among the plants tested, *Acalypha fruticosa, Peltophorum pterocarpum, Toddalia asiatica*,*Cassia auriculata*, *Punica granatum *and *Syzygium lineare *were most active. The highest antifungal activity was exhibited by methanol extract of *Peltophorum pterocarpum *and *Punica granatum *against *Candida albicans*.

**Conclusion:**

This study evaluated the antimicrobial activity of the some ethnomedicinal plants used in folkloric medicine. Compared to hexane extract, methanol extract showed significant activity against tested organisms. This study also showed that *Toddalia asiatica*, *Syzygium lineare*, *Acalypha fruticosa *and *Peltophorum pterocarpum *could be potential sources of new antimicrobial agents.

## Background

According to World Health Organization (WHO) more than 80% of the world's population relies on traditional medicine for their primary healthcare needs. Use of herbal medicines in Asia represents a long history of human interactions with the environment. Plants used for traditional medicine contain a wide range of substances that can be used to treat chronic as well as infectious diseases. A vast knowledge of how to use the plants against different illnesses may be expected to have accumulated in areas where the use of plants is still of great importance [1]. The medicinal value of plants lies in some chemical substances that produce a definite physiological action on the human body. The most important of these bioactive compounds of plants are alkaloids, flavanoids, tannins and phenolic compounds [2].

Rural communities, in particular paliyar tribes, depend on plant resources mainly for herbal medicines, food, forage, construction of dwellings, making household implements, sleeping mats, and for fire and shade. The use of medicinal plants as traditional medicines is well known in rural areas of many developing countries [3,4]. Traditional healers claim that their medicine is cheaper and more effective than modern medicine. In developing countries, low-income people such as farmers, people of small isolate villages and native communities use folk medicine for the treatment of common infections [5].

We chose eighteen plant species used in folk medicine to determine their antimicrobial activity (Table 1). In general, these plants are used in folk medicine in the treatment of skin diseases, venereal diseases, respiratory problems and nervous disorders. Properties of the collected plants are also provided in same table. Evidently, there are not many scientific studies that confirm the antimicrobial properties for most of the plants collected for this study. The phytochemical research based on ethnopharmacological informations is generally considered an effective approach in the discovery of new anti-infective agents from higher plants [6].

**Table 1:** Uses and properties of ethnomedicinal plants collected for antimicrobial screening

Botanical name and Family	Local Name	Mode of preparation, parts used and Ethnomedicinal uses	Properties [26-28]
*Acalypha fruticosa *Forsskal Euphorbiaceae	Chinni chedi	Decoction of leaves prepared in water is taken internally to treat dysentery. Root and leaf paste is prepared in water and applied externally to treat skin diseases.	Stomachic and attenuate
*Albizia procera *Benth. Mimosaceae	Usila maram	Leaf powder is mixed with coconut oil and applied on hair for better growth.	Insecticide
*Cassia alata *L. Caesalpiniaceae	Seemai agathi	Root powder is mixed with lime juice and applied topically on the affected places to treat skin diseases.	Antiparasitic, astringent, purgative
*Cassia auriculata *L. Caesalpiniaceae	Avaarai	Whole plant is powdered with the leaves and stem of *Tinospora cordifolia *and mixed with cow's milk and taken internally to treat diarrhoea. Flowers are crushed and mixed with goat's milk and taken orally to treat venereal diseases	Conjunctives, refrigerant, anthelmintic, stomachic
*Cassia fistula *L. Caesalpiniaceae	Sarak-kondrai	Leaf paste is prepared in water and is taken internally with sugar to treat stomachache. Stem bark and leaves are mixed with coconut oil and made into a paste and applied topically over the affected places to treat skin diseases	Astringent, purgative, anthelmintic, diuretic, laxative, expectorant, anti-inflammatory, cathartic
*Diospyros ebenum *Koenig. Ebenaceae	Beedi elai chedi/Karunthumbai	Fruit powder is mixed with honey and the fruits of *Trichopus zeylanicus*, *Terminalia bellirica*, *Phyllanthus emblica *and rhizome of *Curculigo orchioides *and taken orally to stimulate body stamina.	Astringent
*Diotacanthus albiflorus Benth*. Acanthaceae	Kodi-urinchi	Leaf paste is prepared in water and is applied topically on affected places of wounds and on heel to treat heel cracks.	Bitter
*Elephantopus scaber *L. Asteraceae	Aanai chuvadi	Powder of whole plant is mixed with the leaves of *Toddalia asiatica, Vitex negundo *and *Naravelia zeylanica *and applied externally to treat rheumatism.	Cardiac tonic, febrifuge, astringent and alternative
*Olax scandens *Roxb. Olacaceae	Kaattu pavalam	Decoction of stem bark prepared in water and is taken internally to treat fever and cough.	Febrifuge
*Pavetta indica *L. Rubiaceae	Pavattai	Root powder and dried fruits of *Piper nigrum *are mixed with water and taken internally to get relief from indigestion.	Laxative, tonic
*Peltophorum pterocarpum *(DC.) Backor ex.K. Heyne. Fabaceae	Malai porasu	Paste of stem bark prepared in water is applied topically to treat wounds.	Alternative
*Pterolobium hexapetalum *(Roth.) Sans & Wagh. Fabaceae	Karu indu	Decoction of leaves prepared in water is taken orally by pregnant women during delivery to reduce delivery pain.	Tonic
*Punica granatum *L. Punicaceae	Maadhulai	Dried fruit coat is ground and mixed with water and taken internally to treat stomachache and diarrhoea.	Astringent, anthelmintic, aphrodisiacs, laxative, diuretic, stomachic, cardio tonic, refrigerant
*Solanum xanthocarpum *Burm.f. Solanaceae	Kandan-kathiri	Dried or fresh fruits are kept in fire and the smoke is inhaled through mouth to treat tooth ache Leaf paste prepared in water is applied topically on forehead to get relief from headache.	Pungent, digestive, astringent, carminative, diuretic, expectorant, febrifuge
*Syzygium cumini *Skeels. Myrtaceae	Naval pazham	Leaf juice is mixed with honey or milk and taken orally to treat diabetes. Young leaves are mixed with goat's milk and ground into a paste and taken internally to treat indigestion. Fresh fruits are taken orally to treat stomachache.	Astringent, stomachic, diuretic, tonic and anti-diabetic
*Syzygium lineare *Wall. Myrtaceae	Malai naaval	Fruit powder prepared in water is made into paste and taken internally with the fruits of *Ficus racemosa*, leaves of *Hybanthus enneaspermus*, rhizome of *Curculigo orchioides, Caryota urens *& seeds of *Cissus quandrangularis *to strengthen the body.	Diuretic, stomachic, tonic and astringent
*Tabernaemontana heyneana *Wall. Apocynaceae	Kundalam paalai	Leaf and stem powder is mixed with the stem bark of *Ficus racemosa, Ficus benghalensis, Madhuca longifolia, Strychnos nux-vomica *and fruits of *Carica papaya *and taken internally to induce abortion and applied externally to treat skin diseases.	Anthelmintic and narcotic
*Toddalia asiatica *Pers. Solanaceae	Milagaranai	Leaf paste prepared in water is taken internally to treat stomachache. Powder of bark from stem is used as tooth powder and also used to treat toothache.	Aromatic-tonic, stimulant, antiperiodic, pungent, stomachic

There are a few reports on the use of plants in traditional healing by either tribal people or indigenous communities of Tamil Nadu [7-11]. The development of drug resistance in human pathogens against commonly used antibiotics has necessitated a search for new antimicrobial substances from other sources including plants [12]. Screening of medicinal plants for antimicrobial activities and phytochemicals is important for finding potential new compounds for therapeutic use. This paper reports the results of a survey that was done based on folk uses by traditional practitioners in Palni hills of Tamil Nadu along with bioassay test for antimicrobial activity.

## Methods

### Ethnobotanical survey

Plants were selected for this study based on their medicinal use. Fresh plant parts were collected from the tribal villages (Paliyar tribe) in Palni hills of Tamil Nadu, India in Jan – April 2005. The tribal villages were approximately lie between 10° 12' – 10° 15' N longitudes and 77° 26' – 77° 33' latitude. The ethnobotanical data (local name, mode of preparation, medicinal uses) were collected through questionnaire, interviews and discussions among the tribal practitioners in their local language (Tamil). The voucher specimens in duplicate were deposited in the herbarium of Entomology Research Institute, Loyola College, Chennai (India).

### Preparation of hexane and methanol extract

Plant extracts were prepared by cold percolation method. The plant materials were dried under shade and ground into fine powder using electric blender. 50 g of dried powder was soaked in 300 ml hexane for 48 hours with intermittent shaking. The plant extracts were filtered through Whatman No. 1 filter paper into pill vials. The filtrates were dried until a constant dry weight of each extract was obtained. The residues were stored at 4°C for further use. The remaining plant residue was dried and soaked in 300 ml of methanol as above and the extract was collected as described earlier.

### Antimicrobial screening

The hexane and methanol extracts of 18 plants were screened against a total of 9 bacterial strains and one fungal strain. The test organisms were *Bacillus subtilis *(ATCC 441), *Staphylococcus aureus *(ATCC 25923), *Staphylococcus epidermidis *(MTCC 3615), *Enterococcus faecalis *(ATCC 29212), *Escherichia coli *(ATCC 25922), *Klebsiella pneumonia *(ATCC 15380), *Pseudomonas aeruginosa *(ATCC 27853), *Ervinia sp *(MTCC 2760), *Proteus vulgaris *(MTCC 1771) and one fungal strain *Candida albicans *(MTCC 227) obtained from the Christian Medical College, Vellore, Tamil Nadu.

### Preparation of inoculum

Stock cultures were maintained at 4°C on slopes of nutrient agar. Active cultures for experiments were prepared by transferring a loopful of cells from the stock cultures to test tubes of Mueller-Hinton broth (MHB) for bacteria and Sabouraud dextrose broth (SDB) for fungi that were incubated without agitation for 24 hrs at 37°C and 25°C respectively. The cultures were diluted with fresh Mueller-Hinton and Sabouraud dextrose broth to achieve optical densities corresponding to 2.0·10^6 ^colony forming units (CFU/ml) for bacteria and 2.0·10^5 ^spore/ml for fungal strains.

### Antimicrobial susceptibility test

The disc diffusion method [13] was used to screen the antimicrobial activity. In vitro antimicrobial activity was screened by using Mueller Hinton Agar (MHA) obtained from Himedia (Mumbai). The MHA plates were prepared by pouring 15 ml of molten media into sterile petriplates. The plates were allowed to solidify for 5 minutes and 0.1 % inoculum suspension was swabbed uniformly and the inoculum was allowed to dry for 5 minutes. The different concentrations of extracts (1.25, 2.5 and 5 mg/disc) were loaded on 6 mm sterile disc. The loaded disc was placed on the surface of medium and the compound was allowed to diffuse for 5 minutes and the plates were kept for incubation at 37°C for 24 hrs. At the end of incubation, inhibition zones formed around the disc were measured with transparent ruler in millimeter. The same procedure was followed for the fungus also. These studies were performed in triplicate.

## Results and discussions

Table 1 provides the botanical name, family, local name, plant parts used together with their traditional therapeutic uses and properties for the 18 ethnomedicinal plants collected from Palni hills of Tamil Nadu. Out of the 18 plants tested for antimicrobial activity, 10 plant species showed antimicrobial activity by inhibiting one or more microorganisms. The results of the antimicrobial screening of the crude extracts of all species of plants are shown in Table 2. Among the plants screened, *Acalypha fruticosa, Peltophorum pterocarpum, Toddalia asiatica*,*Cassia auriculata*, *Syzygium cumini *and *Syzygium lineare *showed promising activity against tested microorganisms. The tested plant extracts were most active against gram-positive microorganisms than gram-negative microorganisms. This is in agreement with previous reports by the several workers [14-19].

**Table 2:** Antimicrobial activity of the hexane and methanol extracts of collected ethnomedicinal plants

Plant name	Solvent	Conc. (mg/disc)	Zone of inhibition (mm)									
			
			Bs	Sa	Se	Ef	Ec	Pa	Kp	Es	Pv	Ca
*Acalypha fruticosa *(Aerial parts)	H	1.5	10	9	11	-	-	10	-	-	-	-
		2.5	13	12	13	-	-	13	-	-	-	-
		5	15	14	15	-	-	15	-	-	-	-
	M	1.5	-	-	-	-	-	-	-	-	-	-
		2.5	-	-	-	-	-	-	-	-	-	
		5	-	-	-	-	-	-	-	-	-	-
*Albizia procera *(Stem bark)	H	1.5	-	-	-	-	-	-	-	-	-	-
		2.5	-	-	-	-	-	-	-	-	-	-
		5	-	-	-	-	-	-	-	-	-	-
	M	1.5	-	9	9	-	-	-	-	-	-	-
		2.5	10	11	11	10	-	-	-	-	-	-
		5	12	13	13	13	-	-	-	-	-	-
*Cassia alata *(Leaf)	H	1.5	-	-	-	-	-	-	-	-	-	-
		2.5	-	-	-	-	-	-	-	-	-	-
		5	-	-	-	-	-	-	-	-	-	-
	M	1.5	-	-	-	-	-	-	-	-	-	-
		2.5	-	-	-	-	-	-	-	-	-	-
		5	9	10	-	-	-	-	-	-	-	-
*Cassia auriculata (Leaf)*	H	1.5	-	-	-	-	-	-	-	-	-	-
		2.5	-	-	-	-	-	-	-	-	-	-
		5	-	-	-	-	-	-	-	-	-	-
	M	1.5	-	-	-	-	-	-	-	-	-	-
		2.5	-	9	-	-	-	-	-	-	-	-
		5	10	12	10	10	-	-	-	-	-	-
*Cassia auriculata (Flower)*	H	1.5	-	-	-	-	-	-	-	-	-	-
		2.5	-	-	-	-	-	-	-	-	-	-
		5	-	-	-	-	-	-	-	-	-	-
	M	1.5	-	-	-	-	-	-	-	-	-	-
		2.5	10	11	11	9	-	-	-	-	-	-
		5	13	14	13	12	10	-	-	-	-	-
*Olax scandens *(Leaves)	H	1.5	-	-	-	-	-	-	-	-	-	-
		2.5	-	-	-	10	-	9	-	-	-	-
		5	8	-	-	13	-	12	9	-	-	-
	M	1.5	-	-	-	-	-	-	-	-	-	-
		2.5	-	-	-	-	-	-	-	-	-	-
		5	-	-	-	-	-	-	-	-	-	-
*Peltophorum pterocarpum *(Flower)	H	1.5	-	-	-	-	-	-	-	-	-	-
		2.5	-	-	-	-	-	-	-	-	-	-
		5	-	-	-	-	-	-	-	-	-	-
	M	1.5	9	-	-	-	-	-	-	-	-	9
		2.5	12	10	11	9	-	10	9	-	10	11
		5	14	13	13	12	-	13	12	-	13	13
*Punica granatum *(Root)	H	1.5	-	-	-	-	-	-	-	-	-	-
		2.5	-	-	-	-	-	-	-	-	-	-
	M	1.5	11	12	10	-	-	-	-	-	-	9
		2.5	14	15	12	8	8	-	-	-	-	12
		5	18	19	14	9	9	-	-	-	-	15
*Syzygium cumini *(Seed)	H	1.5	-	-	-	-	-	-	-	-	-	-
		2.5	-	-	-	-	-	-	-	-	-	-
		5	-	-	-	-	-	-	-	-	-	-
	M	1.5	-	11	10	-	-	-	-	-	-	-
		2.5	10	15	13	-	-	-	10	-	-	-
		5	13	18	16	-	-	-	13	-	-	-
*Syzygium lineare *(Leaves)	H	1.5	8	-	-	-	-	8	-	-	-	-
		2.5	10	-	-	-	-	11	-	-	-	-
		5	12	-	-	-	-	14	-	-	-	-
	M	1.5	-	-	-	-	-	-	-	-	-	
		2.5	8	8	10	8	-	8	-	-	8	-
		5	10	10	13	10	-	10	-	-	10	-
*Toddalia asiatica *(Leaves)	H	1.5	-	17	13	-	-	-	-	-	-	-
		2.5	-	23	17	-	-	-	-	-	-	-
		5	-	30	20	-	-	-	-	-	-	-
	M	1.5	-	-	-	-	-	-	-	-	-	-
		2.5	8	10	12	-	-	-	-	-	-	-
		5	10	15	16	-	-	-	-	-	-	-

H – Hexane; M – Methanol-, No activity

Bs – *Bacillus subtilis*; Sa – *Staphylococcus aureus*; Se – *Staphylococcus epidermidis*; Ef – *Enterococcus faecalis*; Ec – *Escherichia coli*; Pa – *Pseudomonas aeruginosa*; Kp – *Klebsiella pneumonia*; Es – *Eruvinia sp*; Pv – *Proteus vulgaris*; Ca – *Candida albicans*.

Methanol extracts exhibited a higher degree of antimicrobial activity as compared with hexane extracts. Methanol extracts of *Albizia procera*,*Cassia auriculata *(leaves and flowers), *Peltophorum pterocarpum*, *Punica granatum *and *Syzygium cumini *showed activity. *Punica granatum *possesed 25% of tannin [20] and the antibacterial activity may be indicative of the presence of some metabolic toxins or broad-spectrum antibiotic compounds. Also ethanolic extract of *Punica granatum *was most active against *E. coli*. Prasanth et al [21] reported that, different extracts of *Punica granatum *fruit showed some antibacterial activity against *P.vulgaris *and *B.subtilis*. Rajakaruna et al [22] reported that *Syzygium cumini *showed good activity against *Staphylococcus aureus *and *Bacillus subtilis*.

Both hexane and methanol extracts of *Syzygium lineare *and *Toddalia asiatica *showed antimicrobial activity. The essential oils from the leaves of *Toddalia asiatica *were most active against *E. coli*, *K. pneumoniae*, *P. aeruginosa *and *S. aureus *[23]. *Peltophorum pterocarpum *and *Syzigium lineare *had the highest inhibitory activity against both gram positive and gram negative bacteria. On the other hand, *Cassia alata *showed only slight activity against bacteria such as *S. aureus *and *B*. *subtilis*. In the previous findings leaves, flowers, root and stem barks of *Cassia alata *showed a range of activity against several bacteria and protozoa [24]. In this study methanol extract of leaves of *Cassia alata *showed antibacterial activity against *Bacillus subtilis *and *Staphylococcus aureus*. Somchit et al [25] also tested the whole plant parts of *Cassia alata *and showed activity in the leaves against *Staphylococcus aureus*.

*Syzygium lineare*, *Punica granatum*, *Syzygium cumini *and *Toddalia asiatica *produced the largest zones of inhibition against *Bacillus subtilis*, *Staphylococcus aureus *and *Staphylococcus epidermidis*. Voravuthikunchai et al [20] reported good antibacterial activity in *P. pterocarpum *and *P. granatum *against *Escherichia coli *using aqueous and methanol extracts. Methanol extracts of *Peltophorum pterocarpum *and *Punica granatum *showed activity against *Candida albicans*. In general, among the tested microbial strains, bacteria were found to be more sensitive to many of the test agents than fungi.

The most sensitive bacterium was *Bacillus subtilis*, which was inhibited by methanol or hexane of 10 plants. On the other hand, no inhibition was observed in the *Eruvinia *sps. Some organisms exhibited only slight susceptibility. *E. coli *was inhibited by methanol extract of flowers of *Cassia auriculata *and hexane extract of *Punica granatum*. *Proteus vulgaris *was inhibited by methanol extract of *Peltophorum pterocarpum *and *Syzigium lineare*. *Klebsiella pneumonia *was inhibited by hexane extract of *Olax scandens*, methanol extracts of *Peltophorum pterocarpum *and *Syzigium cumini*.

## Conclusion

The processing of the plants performed in this study was not comparable to the traditional approach when the Paliyar tribe used water for extracts whereas we have used hexane and methanol for extraction. In this sense it is not an exact replication of the traditional knowledge. All the same, given that methanol extracts were more effective then hexane extract, it is likely that water extracts were will be effective as well and possibly mort so.

The antibacterial activity of *Syzigium lineare*, *Olax scandens*, *Albizia procera *and *Acalypha fruticosa *are reported for the first time. No previous report on the antibacterial activity of these species could be found in the literature. Among the medicinal plants tested in this work, *Peltophorum pterocarpum *and *Punica granatum *showed the most promising antimicrobial properties indicating the potential for discovery of antibacterial principles. Further phytochemical studies are required to determine the types of compounds responsible for the antibacterial effects of these species. The results also indicate that scientific studies carried out on medicinal plants having traditional claims of effectiveness might warrant fruitful results. Several plants used by Paliyar tribe exhibit some degree of antibacterial activity towards gram-positive bacteria such as, *Bacillus subtilis*, *Staphylococcus aureus *and *Staphylococcus epidermidis*. These plants could serve as useful sources for new antimicrobial agents.

## Competing interests

The author(s) declare that they have no competing interests.

## Authors' contributions

VD has carried out the experimental part such as extraction, inoculum preparation and antimicrobial evaluation. MA collected the ethnomedicinal plants from the Palni hills of Tamil Nadu with the help of tribal practitioners. SI supervised the work, evaluated the results and corrected the manuscript for publication. All authors read and approved the final manuscript.

## Pre-publication history

The pre-publication history for this paper can be accessed here:


